# On the ethical governance of swarm robotic systems in the real world

**DOI:** 10.1098/rsta.2024.0142

**Published:** 2025-01-30

**Authors:** Alan F. T. Winfield, Matimba Swana, Jonathan Ives, Sabine Hauert

**Affiliations:** ^1^ Bristol Robotics Laboratory, University of the West of England, Bristol BS16 1QY, UK; ^2^ School of Engineering Mathematics and Technology, University of Bristol, Bristol BS8 1TW, UK; ^3^ Centre for Ethics in Medicine Bristol Medical School, University of Bristol, Bristol BS8 1TW, UK; ^4^ Bristol Robotics Laboratory, School of Engineering Mathematics and Technology, University of Bristol, Bristol BS8 1TW, UK

**Keywords:** swarm intelligence, swarm robotics, responsible robotics, ethical governance, ethical risk assessment

## Abstract

In this paper, we address the question: what practices would be required for the responsible design and operation of real-world swarm robotic systems? We argue that swarm robotic systems must be developed and operated within a framework of ethical governance. We will also explore the human factors surrounding the operation and management of swarm systems, advancing the view that human factors are no less important to swarm robots than social robots. Ethical governance must be anticipatory, and a powerful method for practical anticipatory governance is ethical risk assessment (ERA). As case studies, this paper includes four worked examples of ERAs for fictional but realistic real-world swarms. Although of key importance, ERA is not the only tool available to the responsible roboticist. We outline the supporting role of ethical principles, standards, and verification and validation. Given that real-world swarm robotic systems are likely to be deployed in diverse ecologies, we also ask: how can swarm robotic systems be sustainable? We bring all of these ideas together to describe the complete life cycle of swarm robotic systems, showing where and how the tools and interventions are applied within a framework of anticipatory ethical governance.

This article is part of the theme issue ‘The road forward with swarm systems’.

## 1. Introduction

Swarm robotics is a growing approach to the coordination of multi-robot systems. Unlike traditional multi-robot systems that use centralized or hierarchical control and communication systems to coordinate robots’ behaviours, swarm robotics adopts a decentralized approach. In this approach the desired collective behaviours emerge from the local interactions between robots and their environment. Such emergent or self-organised collective behaviours are inspired by—and in some cases directly modelled on—the swarm intelligence observed in social insects.

The potential for swarm robotics is considerable. Any task in which physically distributed objects need to be explored, surveyed, collected, harvested, rescued or assembled into structures is a potential real-world application for swarm robotics. Application domains include search and rescue, precision agriculture, environmental monitoring, logistics and—using nanorobots—cancer treatment. The key advantage of the swarm robotics approach is robustness, which manifests itself in a number of ways. First, because a swarm of robots consists of a number of relatively simple and typically homogeneous robots, which are not pre-assigned to specific roles or tasks within the swarm, then the swarm can self-organise or dynamically re-organise the way individual robots are deployed. Second, and for the same reasons, the swarm approach is highly tolerant to the failure of individual robots. Third, control is completely decentralized, so there is no common-mode failure point or vulnerability in the swarm. Indeed, it could be said that the high level of robustness evident in robotic swarms comes for free in the sense that it is intrinsic to the swarm robotics approach. This is in contrast with the high engineering cost of fault tolerance in conventional robotic systems.

To the best of our knowledge, there are—at the time of writing—no real-world commercial deployments of swarm robotic systems. However, a number of real-world applications have been demonstrated. The survey by Schranz *et al*. [1] outlines demonstrations of swarms across a range of applications, usually demonstrated as part of research projects. Another recent paper by Dorigo *et al*. [2] puts the real-world use of robot swarms in agriculture and inspection, military information gathering and mission support, maritime and deep-sea applications and ecological monitoring by 2030, as well as the deployment of swarms in cities within 10 years. Future applications mentioned include space exploration and expansion, collecting microplastics and targeted drug delivery using nanoswarms. Key to this translation is our increased ability to produce more sophisticated low-cost robots that are able to perceive and interact with their local environment using on-board sensing, processing and intelligence [3].

If we adopt the view, not unreasonably, that swarm robotic systems will be applied in the real-world within a decade [2], then we should be asking the question: how can we ensure that such systems are designed, built, deployed and operated responsibly? This paper addresses that question by setting out a set of principles, tools and practices for the ethical design and deployment of swarm robotic systems. These principles, tools and practices should, we argue, be applied within a framework of ethical governance.

At this point we should pause and address the important question: given that comprehensive frameworks for values-driven design and ethical governance have already been set out for non-swarm robotics and artificial intelligence (AI) systems [4–9], why are these not sufficient to cover swarm robotic systems? Or, to put it another way, what is it about swarm systems that require us to go beyond existing approaches to responsible robotics? The short answer is *emergence*. In conventional engineered systems, including single- and multi-robot systems, an emergent property is a bug, which must be fixed. In swarm systems emergence is not a bug but a desired property. Designing swarm systems that harness self-organisation and emergence is challenging, not least because swarm systems are typically stochastic [10].

A second notable property of swarm robotic systems is the unusually high level of autonomy of the individual robots of the swarm. Typically, individual robots will decide their own next actions based only on local sensing and, perhaps also, communication with neighbouring robots. Yet, given the clear risks of multiple physical robots interacting with humans or the environment, it is essential that real-world swarm robotic systems are subject to human monitoring, oversight and, if necessary, intervention. Underpinning this is our view that real-world swarm robotic systems are *socio-technical* systems and, in this respect, are no different to social robots such as care robots or smart robot toys.

A third property relates to the scale at which swarms are deployed, involving tens, hundreds, thousands or even millions of robots (for nanomedicine). Such scale, combined and often requiring autonomy of the individual robots, means new approaches to understand, monitor and control large collectives of robots. Recognition of the need for human oversight of swarm systems has given rise to the field of human–swarm interaction,^1^ with necessarily different approaches to conventional human–robot interaction [11,12].

The unusual demands of emergence and human–swarm interaction do not mean that swarm systems cannot be engineered for safety and dependability, but the approaches required are different. Recent work by Assaad & Boshuijzen-van Burken [13], for example, has looked at the ethics and safety of human–machine teaming. Existing methods for values-driven design still apply equally, but swarm systems will need an additional layer of consideration of swarm-level risks, mitigation and management.

The key contribution of this paper is a new framework for the ethical governance of swarm robotic systems. In §2, we present a set of new ethical principles that we believe should guide and underpin the design and use of all swarm robotic systems. The section relates these swarm principles to existing ethical principles for non-swarm robotics and autonomous systems, as well as highlight the risks that are unique to swarm engineering. In §3, we introduce the ethical risk assessment (ERA), with reference to British Standard BS8611. ERA is a powerful tool for systematically considering likely risks and how they might be mitigated, and is an important part of our proposed ethical governance framework. In §4 we present in detail the new framework for ethical governance of swarm robotic systems. Then, in §5, we illustrate the work of this paper by developing four original case studies of real-world swarm robotic systems. Section 6 concludes the paper with a discussion of opportunities and challenges in implementing risk mitigations when using swarm technology.

## 2. Ethical principles for swarm systems

In the past 15 years, many sets of ethical principles have been proposed for robotics and AI. Notable among these are the European Commission’s High-Level Expert Group on AI Ethics Guidelines for Trustworthy AI^2^ and the Organisation for Economic Co-operation and Development principles of AI.^3^ Jobin *et al*. [14] surveyed 84 sets of ethical principles, including these two, and found that the 84 frameworks have a great deal in common. Transparency is the principle most frequently included, appearing in 73 out of 84 frameworks. The authors of the study note that no single ethical principle appeared to be common to the entire corpus, although there is an emerging convergence around transparency, justice and fairness, non-maleficence, responsibility, privacy and beneficence. It is important to note that the principle of non-maleficence, or ‘do no harm’, is mirrored by beneficence, or ‘do good’. Beneficence should be a key requirement of responsible robotics; otherwise, systems that are shown to be safe and secure may not provide any positive benefit to society and/or the environment. Umbrello & van de Poel [7] set out a model of values-sensitive design that distinguishes between ‘values promoted by design and values respected by design to ensure the resulting outcome does not simply avoid harm but also contributes to doing good’.

Since the survey by Jobin *et al*. [14], there have been a number of similar works including Hagendorff [15] and Huang *et al*. [16]. Hagendorff [15] is notably critical, concluding that, ‘Currently, AI ethics is failing in many cases. Ethics lacks a reinforcement mechanism. Deviations from the various codes of ethics have no consequences’. Huang *et al*. [16] surveyed a total of 146 AI ethics guidelines published between 2015 and 2021. Of particular value is their comprehensive analysis and critique of the categorisation of ethical issues, leading Huang *et al*. to propose a clear and easy-to-understand classification, with three high-level classes, as listed in table 1.

**Table 1.** Proposed high-level categories of ethical risk, adapted from Huang *et al*. [16].

class	ethical issues
individual	safety and security, privacy and data protection, freedom and autonomy, human dignity
societal	fairness and justice, responsibility and accountability, transparency, surveillance and datafication, controllability of AI, democracy and civil rights, job replacement, human relationships
environmental	natural resources, energy, environmental pollution, sustainability

Hunt & Hauert [17] proposed—for the first time—a set of safety principles for swarm robotic systems, and we make use of these principles as the foundation for ethical governance in this paper. Hunt & Hauert express their swarm principles as a set of 10 issues and questions. These are listed in table 2, alongside—in the fourth column—a mapping on to the general ethical categories of Huang *et al*. [16]. The value of this mapping is that it shows that the 10 principles all map to common ethics principles.

**Table 2.** Keywords and questions from Hunt & Hauert [17] mapped to the high-level ethics principles in table 1. Note that the word *individual* in column 3 refers to individual robots, whereas the same word in column 4 [16] refers to an individual human. In column 4, we also show in parentheses which issues are specific to single robots and which to the swarm as a whole.

	issue	question (from Hunt & Hauert [17])	mapping to Huang *et al*. [16]
1	ethics	is this an ethical use of a robot swarm?	individual, societal and environmental
2	legal	does the swarm comply with all relevant laws and regulations for the domain(s) of deployment?	societal: fairness and justice (swarm)
3	accountability	is there a way to analyse swarm failures?	societal: responsibility and accountability, transparency (swarm)
4	user interaction	can the users interact with the swarm to prevent unwanted behaviour?	societal: controllability (swarm)
5	physical harm from individual robots	can the individual robots cause physical harm to humans, animals or the environment?	individual: safety; environmental (robot)
6	physical harm from the swarm	can the emergent swarm behaviour cause physical harm to humans, animals or the environment?	individual: safety; environmental (swarm)
7	behavioural harm from individual robots	can the behaviour of individual robots result in unsafe operation?	individual: safety (robot)
8	behavioural harm from the swarm	can failure of the emergent swarm behaviour cause unsafe operation?	individual: safety (swarm)
9	security of individual robots	can individual robots be maliciously hacked?	individual: security (robot)
10	security of the swarm	can the emergent swarm behaviour be subverted by malicious actors?	individual: security (swarm)

We can make a number of observations from table 2. The first is that principle 1, *Ethics,* is really a meta-level principle, as the question ‘is this an ethical use of a robot swarm?’ encompasses all three classes in table 1. Our second observation is the separation of swarm-level issues and questions 1, 2, 3, 4, 6, 8 and 10, as well as single robot issues and questions 5, 7 and 9. This principled separation underpins much of the work of this paper.

Consider also the second question in table 2, ‘does the swarm comply with relevant laws and regulations?’^4^ Regulation requires (i) relevant law, (ii) a regulator and (iii) standards against which the regulator requires compliance. Intelligent robots are certainly within the scope of the EU AI Act, which became law in August 2024 [18]. As Ebers [19] puts it, smart robots are ‘AI in action in the physical world’. We must assume, therefore, that swarm robotic systems will fall within the remit of the AI Act and that some real-world swarm systems will be classed as ‘high risk’. For a recent overview of standards and regulations in robotics and AI, see Winfield & Studley [20].

## 3. ERA for swarm robotic systems

Risk assessment is a well-known method for discovering and mitigating risks and hence improving safety. However, the idea of extending the envelope of risk assessment of intelligent systems to encompass ethical risks is relatively new. Ethical Risk Assessment (ERA) offers a powerful method for systematically anticipating and mitigating the ethical, societal and environmental risks associated with the use of robots and AI. We place ERA at the heart of our ethical governance framework for swarm robotic systems.

We base our approach to ERA on British Standard BS8611:2023 *Guide to the ethical design and application of robots and robotic systems* [21]. ‘BS8611 is not a code of practice, but instead guidance on how designers can undertake an ERA of their robot or system, and mitigate any ethical risks so identified. At its heart is a set of 20 distinct ethical hazards and risks, grouped under four categories: societal, application, commercial & financial, and environmental. Advice on measures to mitigate the impact of each risk is given, along with suggestions on how such measures might be verified or validated’ [22].

BS8611 defines an *ethical harm* as ‘anything likely to compromise psychological and/or societal and environmental well-being’. An *ethical hazard* as ‘a potential source of ethical harm’ and an *ethical risk* as the ‘probability of ethical harm occurring from the frequency and severity of exposure to a hazard’. Thus extending the envelope of risk assessment to include ethical harms, hazards and risks (over and above physical harms, hazards and risks).

For example case studies of BS8611-guided ERA of smart robot toys, see Winfield *et al.* [23], and a hospital disinfectant robot, see McGinn *et al.* [24]. The first of those studies concluded that ‘attention to ethical risks can:

— draw attention to potential design modifications to mitigate some risks,
— highlight the need for user engagement,
— reject product functionality as too risky,
— suggest new functions, and/or
— indicate potential future issues, highlighting the need for periodic reassessments’,

while also noting that the ERA is not guaranteed to expose all ethical risks. ‘It is a subjective process which will only be successful if the risk assessment team are prepared to think both critically and creatively about the question: what could go wrong?’ [23]. As Vallor *et al*. [25] observed, design teams must develop the ‘habit of exercising the skill of moral imagination to see how an ethical failure of the project might easily happen, and to understand the preventable causes so that they can be mitigated or avoided’.

The process of ERA, as set out in BS8611, is equally applicable to swarm robotic systems as it is to single robots. The one difference is that ERA for swarm systems will need to consider hazards, risks and mitigation at the level of *both* individual robots and the swarm system as a whole. This difference is both minor in the sense that the process of ERA is identical and significant in the sense that swarm-level properties are of key importance.

## 4. A framework for ethical governance of swarm robotic systems

All real-world engineered systems progress through multiple stages, from conception through design, test, deployment and, ultimately, end-of-life decommissioning. Swarm robotic systems are no different but require an additional layer of consideration of swarm-level risks, mitigation and management.

We now set out a framework of anticipatory ethical governance for swarm robotic systems, which embeds the ethical principles of table 2 alongside ERA and ethical oversight. The framework is presented as a flowchart from conception to end of life in two parts: (i) from conception to pre-deployment and (ii) from deployment to end of life.

### (a) From conception to pre-deployment

**Figure 1.**
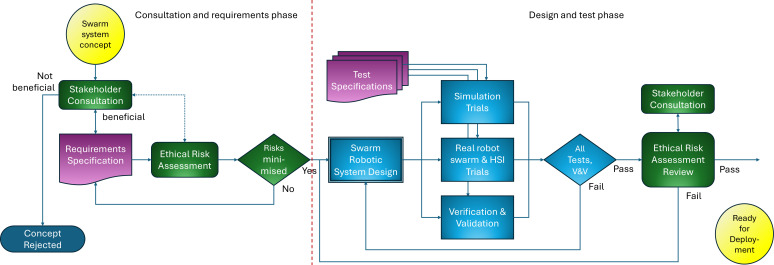
Swarm system lifecycle part 1: from requirements to readiness for deployment. Note that all elements of ethical governance are shown in green.

Figure 1 details part 1 of the swarm system lifecycle, from consultation and requirements through design and test to readiness for deployment.

Consider first the consultation and requirements phase. All engineered systems start as a concept. The concept may arise within a company research department, but for swarm robotic systems, the concept is more likely to have been proposed and demonstrated, either in a university laboratory setting and/or (less often) in the field.

For swarm systems, we propose that the system concept must first be presented to stakeholders for consideration. Depending upon the application, the stakeholder group might consist of, for instance, community representatives, local government officers, lawyers, regulators and representatives of environmental groups, alongside lead engineers and senior managers of the enterprise proposing the system. Here we are borrowing a principle from the integrative social robotics work of Fischer *et al*. [26]: ‘The Quality Principle: The research, design and development process must involve, from the very beginning and throughout the entire process, expertise of all disciplines that are relevant for the description and assessment of the socio-cultural interaction(s) involved in the envisaged application’.

The three stages of the consultation and requirements phase in figure 1 are as follows:

1. Presentation of the swarm system concept to the stakeholder group. The goal of *Stakeholder Consultation* will be to establish whether or not the concept meets the first two principles of table 2, namely *Ethics*, addressing the question: is this an ethical use of a robot swarm? and *Legal*, addressing the question: would the swarm application (at least in principle) comply with all relevant laws and regulations? Given that the answer to the legal question will be fully determined in later stages, then the primary focus should be on whether or not the stakeholder group judge the concept to be ethical and beneficial. It follows that the group may reject the concept altogether.
2. Development of the requirements specification for the proposed swarm robotic system by the swarm system design team. Here we expect that the design team will actively engage with the stakeholder group to ensure that any concerns, such as recommended constraints or controls on the system are reflected in the requirements specification.
3. The requirements specification is subjected to ERA, as outlined in §3. Given that ERA is a subjective exploration of what could go wrong, it should be undertaken by a team drawn from both the design team and the stakeholder group, as experience suggests that a mix of technical and non-technical backgrounds helps to think outside the box [23,25].

Consider now the design and test phase of figure 1. The first stage is the design of the swarm robotic systems. Let us break this down, as there are several layers that need to be co-designed.

— Design of the individual robots of the swarm; their morphology, sensing and actuation. Also, the means by which robots signal to each other and to the operators of the swarm. Careful attention also needs to be given to the robot’s internal power source and how it will be recharged.
— Design of the overall swarm behaviours. Because we are designing for emergence and self-organisation at the swarm level, then design needs a top–down approach—even though what we are designing are the controllers embedded in individual robots. A powerful automated approach is to use a genetic algorithm to evolve robot behaviours. The method developed by Jones *et al*. [27] has the important advantage that the evolved controllers are human-readable behaviour trees.
— Design of the human–swarm interface (HSI) system and its infrastructure. This is a critical subsystem as it provides the means by which the overall swarm can be monitored and managed.
— Design of the subsystems for both launching and recovering the entire swarm. Control of these processes will be part of the swarm HSI, but—given that the swarm is a physical collection of robots—launch and recovery will need physical infrastructure. The same infrastructure will be needed to recover individual robots that need repair or maintenance.
— Depending on the application some swarm systems—in particular those intended to operate continuously—will need an additional infrastructure for robot battery re-charging. This might take the form of charging stations distributed across the operational environment or a centralized charging station to which batches of robots can go for re-charging. In either case, individual robots will monitor their own battery levels and autonomously decide when to break out of the swarm, move either to the nearest free charger or to the centralisation charging station and then return to the swarm when charging is complete.

In parallel with swarm system design, the design team will draft test specifications for three sets of tests: simulation trials, real robot swarm and HSI trials, and verification and validation of both individual robots and the swarm as a whole.

In practice, simulation of the swarm is likely to also be part of the design stage, as simulation tools provide a means for rapid development and testing of swarm designs. However, the three test stages shown in blue in figure 1 are not intended to be part of design but a formal and documented test stage once the design team regards the design as complete. Of course, some tests are likely to fail, in which case the process will iterate back to the design stage.

The final stage of the design and test phase is an ERA review involving the stakeholder group and members of the design team. The purpose of this review is twofold. First, to revisit the ERA of the consultation and requirements phase, paying particular attention to how the design team has realised the mitigations recommended in the initial ERA. Second, to consider the ethical risks of aspects of the design that could not have been anticipated in the consultation and requirements phase; these might include, for instance, the materials used in the design of the robots, the HSI, launch and recovery infrastructure designs and their ethical impacts. If the review team determines either that some recommendations of the initial ERA have not been adequately addressed or that aspects of the design have unacceptable risks that need mitigation, then the review team will require design changes, followed by re-test and re-review.

As we remarked in §2, it is possible that the process of ERA and its focus on risk mitigation might have caused the need also for positive benefits from the swarm system—the requirement of beneficence—to be overlooked during the design and test phases. Ensuring that the societal and/or environmental benefits envisioned in the consultation and requirements phase are not overlooked is also part of the role of stakeholder engagement in this and later phases.

### (b) From fabrication and deployment to end of life

**Figure 2.**
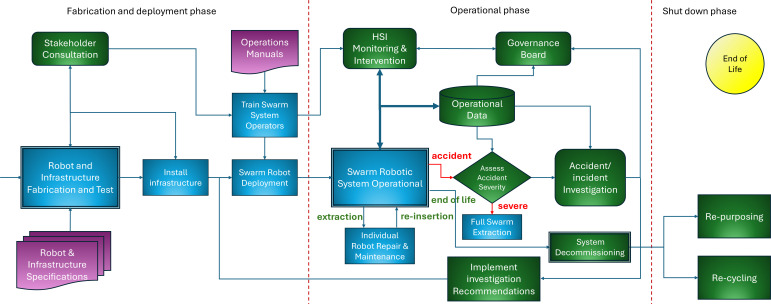
Swarm system lifecycle part 2: from fabrication and deployment to end of life. Note that all elements of ethical governance are shown in green.

Figure 2 details part 2 of the swarm system lifecycle, from fabrication and deployment through operation to shut down and end of life.

Much of the fabrication and deployment phase of figure 2 is self-explanatory. First, the robots of the swarm and infrastructures for both the HSI and robot launch and recovery must be fabricated and tested. Then the infrastructures must be installed and made operational before the swarm robots are deployed. In parallel, the swarm system operational team must be trained. Stakeholder oversight of fabrication and test is needed to provide assurance that tests are thorough and fully documented. Oversight of infrastructure installation is needed to satisfy stakeholders that environmental concerns have been properly addressed. The link between *Stakeholder Consultation* and *Train Swarm System Operators* in figure 2 reflects the need for the operational team to be trained not only on the technical aspects of managing the swarm system but also on the ethics and values that have been embedded in its design. It is important to establish trust and openness between stakeholders, especially community groups, and the operations team so that when issues arise during swarm operation—as they inevitably will—they can be resolved cooperatively and transparently.

We shall now consider the operational phase of figure 2 in the following three sections: normal operation, contingencies and governance.

1. Normal operation: The two key processes of normal operation are *Swarm Robotic System Operational* and *HSI Monitoring & Intervention*. The details of these processes will vary significantly depending on the application, and four examples are outlined in the case studies of §5. An important element of normal operation is the collection of operational data. These data will capture (i) the activity (battery status, sensory inputs, motor outputs and behaviour) of individual robots in the swarm, (ii) the disposition of robots in the swarm as a whole, and (iii) any interventions by the operating team. The data are needed for the following two reasons: (i) to support the compilation of post hoc reports of normal swarm operation for both the operations team and the operations oversight board, and (ii) to provide key data in the event of unexpected behaviours, incidents (near-miss accidents) and accidents.
2. Contingencies: Broadly there are two kinds of contingencies: (i) individual robots needing repair and/or maintenance, and (ii) swarm-level accidents, incidents or simply unexpected or unintended behaviours. The first of these is shown in the *Individual Robot Repair and Maintenance* process of figure 2, showing extraction of the robot that needs attention, then re-insertion following repair. The second contingency is potentially much more serious. In the event of an accident that causes harm, then the operations team may need to trigger a full swarm extraction—shutting down swarm operations—followed by an investigation. Following a near-miss incident,^5^ or if the operations team notice something unusual or unexpected they will need to determine whether or not it is safe to investigate while the swarm continues to operate (and protocols will need to be designed to support this decision).^6^ *Accident/Incident Investigation* draws upon both operational data logs and witness testimony and seeks to address three questions: (i) (factual) what happened (or nearly happened)? (ii) (explanatory) why did it happen? and (iii) (practical) what can we do to ensure it does not happen again? [29]. To date, there has been no work on accident investigation focused explicitly on swarm robotic systems, but recent work on accident investigation for social robots would provide a good starting point [30,31]. Accident investigation needs to be undertaken by an independent team appointed by the *Governance Board* (see below). Once the accident/incident investigation has concluded, its recommendations must be implemented prior to redeployment of the swarm robotic system and resumption of operation. It is important to note that those recommendations might require design changes to the individual robots, the HSI, the various system infrastructures and/or operational procedures.
3. Governance: An essential element of ethical governance is continuous monitoring and oversight, and here we propose a governance board charged with that responsibility. Importantly, the governance board will not be merely advisory or consultative but have the power to require operational changes should the board determine that there are serious safety issues or that ethical values and principles have been compromised. The board’s membership should, we propose, include (i) representatives of each of the key stakeholder groups already engaged in earlier phases, (ii) the operational team leader—who will be the primary channel of communication between the board and the operations team. Other specialist members of the operations team could be called in as needed. And (iii) one or more independent experts in the application domain—depending on the level and complexity of risk. One of these independent experts would be called upon to chair the board. It is important also that the chair has the confidence of both the stakeholder group and the operations team. The level of oversight of the governance board is likely to change during the operational lifetime of the swarm robotic system. During an initial ‘probationary period’, the governance board would be highly proactive in monitoring swarm operations. Figure 2 shows a direct link between the operational data repository, as we would expect the governance board to have direct access via a ‘dashboard’, which would provide summary reports and visualisations—as well as the option to drill down into the data logs. The board would decide—on the basis of the frequency of unexpected or unusual swarm behaviours—when the probationary period can be concluded, following which the board would switch to a lighter-touch oversight mode. In the event of serious accidents or incidents, the board would be responsible for initiating the accident investigation,^7^ which would report its findings and recommendations back to the board. Following such an investigation, the level of oversight by the governance board should switch back to probationary mode.

The final shut down phase shown in figure 2 shows two options following decommissioning: re-purposing and re-cycling. Of the two, re-purposing of the robots and (ideally) supporting infrastructure is by far the most sustainable [32]. But if re-purposing is not viable, then the responsible re-cycling of materials and components is essential.

In summary, our framework is built upon a conventional engineering lifecycle but adds a layer of additional ethical governance that comprises six processes: (i) stakeholder consultation and governance, (ii) ERA, (iii) human–swarm interaction for monitoring and intervention, (iv) data logging, (v) accident/incident investigation and (vi) end-of-life re-purposing and/or re-cycling.

## 5. Four use case studies

**Figure 3.**
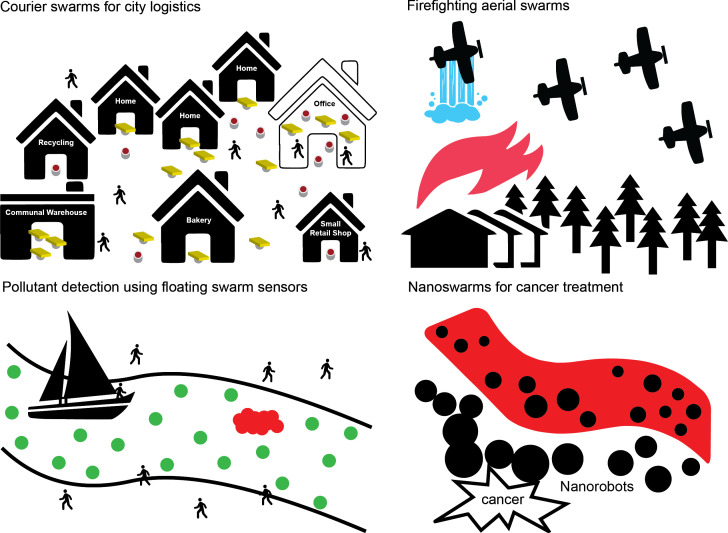
Illustrative depictions of the four case studies: (1) Courier swarms for city logistics, (2) Firefighting aerial swarms, (3) Pollutant detection using floating swarm sensors and (4) Nanoswarms for cancer prevention.

To illustrate the need for ethical governance, we focus on the following four representative and realistic case studies in swarm robotics: (i) courier swarms for city logistics, (ii) firefighting aerial swarms, (iii) pollutant detection using floating swarm sensors and (iv) nanoswarms for cancer prevention. Depicted in figure 3, these were chosen as they vary in scale (number of robots), how safety-critical they are and their environments (public spaces, aerial spaces and the human body). For each, we highlight ethical risks resulting from the swarm properties of the system (e.g. emergence, distributed control, scale, robustness and adaptability). The aim is not to provide a thorough ethical analysis for each but to highlight salient issues.

### (a) Courier swarms for city logistics

Scenario: Courier robots, owned by local communities (e.g. buildings and neighbourhoods), are readily available in the environment (e.g. every floor or every street corner). The ground-based robots can be called to pick up an item and deliver it to a desired location within the community. When large distances need to be travelled, robots coordinate with nearby community robots to pass on items. Items carried include package deliveries, pastries from the local coffee shop and item swaps with neighbours [3].

Stakeholders: local communities, city councils, local shops and delivery companies.

Table 3 lists an outline of an ERA of a swarm robot courier system for local communities. A more detailed analysis of the risks and mitigations follows below.

**Table 3.** ERA for the deployment of swarm couriers in local communities. Risk levels (estimated likelihood) are assessed as high (H), medium (M) and low (L). The categories in column 1 are from m Huang *et al*. [16], table 1.

category	ethical risk	level	mitigation
individual	collisions or obstructions with important throughways	M	robots should be visible/audible, small enough to get around and avoid aggregation.
individual	swarm collects sensitive information from communities	M	use on-board processing of information relevant for the robots’ operations only (edge computing) and regularly delete data collected by the robot.
individual	swarm is tasked with illegal operations (e.g. transporting drugs)	M	require identification of senders and random package checks by local authorities (e.g. using a master key).
societal	lack of trust from local communities	H	co-design, ownership and control of swarms by local communities.
societal	impact to employment	M	focus the swarm on unmet needs (hand-to-hand deliveries), favour human roles with human interaction and allow users to ask for human delivery.
societal	inequality of access to technology	H	government investment/subsidies towards the deployment of robots in every community.
societal	consent not granted to receiving goods	L	consent required before sending a robot on delivery with limits on the number of deliveries made. Content of the robot should be visible to the receiver.
societal	lack of clarity on who operates/owns the swarm	M	ownership and contact information should be visible on each robot.
environmental	swarm design is not sustainable	M	favour sustainable production, easy maintenance and re-cycling of material, edge computing and integration of robots in the environment.
environmental	overconsumption leads to environmental impact	M	favour local circular economies, enabling exchange/sharing of goods and local businesses.

#### (i) Individual risks

Harm by individual robots and the swarm: Robots may collide with people, animals or vehicles and could cause tripping hazards. Care will need to be taken to make the robots visible when in operation (bright flags that identify the robot operating on the ground). Priority should be given to making sure individual robots stop in case of potential collision and have alert sounds when objects are too close. They could also be designed to be small and lightweight. Finally, the swarm itself could cause issues, for example, by aggregating on roads or areas of importance (e.g. hospital entrances). Swarm control should be designed to avoid aggregation and resulting obstructions.

Lack of privacy and confidentiality: At scale, the robots through their cameras and microphones will feel obtrusive to local communities. If not carefully managed, they could create high-fidelity maps of the environment and collect protected data about work, home and public environments accessible to the robots. Mitigation will require on-board processing of information relevant for the robots’ operations only (edge computing). Video and images used for operation will need to be deleted as soon as possible after processing.

Misuse: The swarm could be used to transport illegal items, such as drugs. This could be mitigated by requiring the identification of senders and random package checks by local authorities (e.g. using a master key).

#### (ii) Societal risks

Loss of trust: The large-scale deployment of swarms in local communities, if not carefully designed, may lead to a lack of trust in swarms. This could be due to the tasks the robots are asked to carry out (e.g. transporting illegal items) or to the behaviour of the robots (loss of item, malfunctioning, blocking walkways, bike paths or roads). Mitigation will require careful co-design and, possibly, ownership and control of the robots by local communities.

Employment: If delivery swarms are present at scale, they could disrupt the current logistics labour market, with less need for delivery staff to bring items to doors. Jobs may also be displaced from roles that are human facing (e.g. delivery) to management of swarms behind dull interfaces. The swarms may also drive human operations (by tasking humans to complete actions such as loading or unloading the robots). These issues could be mitigated by using courier swarms in areas of unmet need, for example, local transport of items from hand-to-hand (sharing of goods, local shops, in-building transport of goods and redelivery of local packages). The distributed nature of swarms may also give more control to the human operators/local communities in directing a robot completing a task for it (rather than a central interface). Finally, there is the unwritten role of a post-person who chats and takes care of people. Retention of human contact should be nurtured, for example, by allowing the receiver to request a human rather than a robot for their delivery.

Inequality of access: If robots are owned by local communities, they may only be available to those with necessary means, reinforcing inequality of access to goods. Mitigation may take the form of government investment/involvement in the deployment of robots in every community, similar to large-scale deployment of e-scooters. This may, however, require a centralized service-based deployment rather than a community-owned one.

Lack of informed consent: Robots may send unwanted/illegal items to unexpecting receivers. At scale, this could give rise to multiple robots showing up at doors of people; this could also pose a security threat if the payload of the robot is nefarious. Consent should, therefore, always be provided to receive goods before the robot is sent on its delivery mission, and there should be limits on how many deliveries are expected at one time. Furthermore, the payload of the robot should be made visible to the receiver before reception (similar to looking through a door’s eyepiece).

Lack of informed command: As robots are owned by local communities, it may be unclear who is responsible for their operations and deployment. This should be made clear by having the robot support clear markings regarding their community of operation, ownership and how to interact and query the robot.

#### (iii) Environmental risks

Lack of environmental awareness (robots and operations): Every local community owning several robots will require substantial resources in terms of energy, and material use, similar to the production of household electronic appliances. Their need for computation may have energy implications (for example, if images have to be processed off-board through machine learning models). Finally, swarms many transform the cityscape, with robots present in many environments. Mitigation should require sustainable design of the robot, favouring easy maintenance and re-cycling of material. Furthermore, edge computing should limit the amount of learning-based or off-board computation required. Where possible, swarms should be embedded in the environment, perhaps in attractive storage boxes that are well-integrated and visually/environmentally pleasing.

Lack of environmental awareness (application): The ease of transport of goods at a local scale could lead to over consumption and more online deliveries. Mitigation could instead promote local circular economies, sharing of goods and consumption from local producers and shops.

### (b) Firefighting aerial swarms

Scenario: A swarm of 30 large payload UAVs are deployed 24/7 for 3 months over California to detect and mitigate forest fires. The robots can travel 1000 km and carry large extinguishing payloads. They are linked to a small unit called the swarm fire brigade that oversees their operations and informs missions when fires are detected [33].

Stakeholders: governments, firefighters and population at risk of wildfires.

Table 4 lists an outline of an ERA of an aerial swarm for firefighting. A more detailed analysis of the risks and mitigations follows below.

**Table 4.** ERA for the deployment of aerial swarms for firefighting.

category	ethical risk	level	mitigation
individual	collisions, crashes or incorrect drop of extinguishing agents	M	safety first design, including verification and validation of individual aircraft in accordance with existing regulations and safe fallbacks to swarm operation. Recording of data (black box) to allow for investigations.
societal	inappropriate trust in the fire detection and suppression capabilities	M	thorough testing and confidence building in swarm capabilities. Close partnership with firefighters as a single team.
societal	swarms of drones are of concern to people of different backgrounds	M	clear markings/sound related to firefighting to clearly identify their purpose.
societal	inequality of access to technology	H	engaging with international organisations (e.g. the UN-COP) for global operation.
societal	fires are erroneously extinguished without consent	H	human oversight required to engage a fire.
societal	lack of clarity on who operates/owns the swarm	M	clear markings related to firefighting to clearly identify their ownership and operating entity.
societal	swarm is used for surveillance	M	use on-board processing of information relevant for the robots’ operations (edge computing) only transmit information back when it relates to a fire detection and the need for human input—or on demand from a human operator. Delete images processed on-board the robot as soon as possible.
societal	swarm is used for other applications (e.g. warfare)	H	require a separate ERA to determine appropriate alternative/dual uses.
environmental	swarm deployments are not sustainable	L	a full lifecycle assessment should be carried out to understand the environmental cost of swarm deployments (continuous operation with petrol-based robots) versus the positive impact of controlling wildfires.

#### (i) Individual risks

Harm by individual robots and the swarm: Large aerial robots could cause damage or harm if they collide with each other or human environments. Care needs to be taken to verify and validate the operations of individual aircraft before scaling up the numbers deployed. Safe fallback behaviours (such as loitering) should be considered when the swarm behaves in unexpected ways, allowing the system to safely recover. Data should be recorded to allow for further investigation.

Lack of privacy and confidentiality: The swarm, through its ability to image all environments from above, will feel obtrusive if allowed to record and share footage. Mitigation will require on-board processing of information relevant for the robots’ operations to detect fires and to only transmit footage when fires are detected. Video/images irrelevant to firefighting will need to be deleted immediately after processing.

Misuse: The swarm could be used for other applications such as warfare and surveillance. These would need their own ERA to determine their appropriate use.

#### (ii) Societal risks

Loss of/inappropriate trust: Like many safety-critical systems, the continuous deployment of swarms over large areas to detect and mitigate forest fires will need to meet a high threshold of accuracy to avoid the public losing trust in the system. Work on swarm specification, verification and validation could enable this. Until this confidence is built, it will also be important to not overly rely on the system, instead focusing on the swarm as an aid to firefighters.

Lack of respect for culture diversity and pluralism: Firefighting drones may be mistaken for military drones used in warfare. The presence of drones in everyday environments may cause concern for certain communities. It will be important for the robots to carry clear markings related to firefighting, and perhaps even a recognisable sound, to clearly identify their purpose.

Inequality of access: Only countries with the means to operate a firefighting swarm will be able to avoid wildfires and resulting economic, health and environmental impact. This inequality of access could be mitigated by engaging with international organisations (e.g. the United Nations) to operate this as a service globally.

Lack of informed consent: Fires detected may be intentional and non-criminal (e.g. controlled burns and BBQs). These fires may, however, be detected and engaged with automatically. Instead, consent should be granted to attack the fire by either (i) having firefighters monitor the fire feed with the aim of providing human approval of fire mitigation or (ii) allowing members of the public to signal a safe fire through an app or other interaction modes.

Lack of informed command: It may not be clear who is responsible for the command of the swarm or how to engage with it. This could be provided through clear markings and announcements. Ideally, announcements are initiated by the fire brigade operating the swarm so as to show a clear line of command.

#### (iii) Environmental risks

Lack of environmental awareness (robots and operations): Operations may require many large aircraft operating on petrol, over large periods of time. This consumption could be offset by moving to electric vehicles or by making the aircraft more efficient and optimising the deployments, for example, by only flying in high-risk areas at high-risk times. More broadly, the swarm could also be engineered to be useful at all times, even when no fires are present, for example, providing services such as land management, flood mapping or aid delivery in disasters.

Lack of environmental awareness (application): If successful, mitigating wildfires has the potential to offset twice the global carbon emissions from the aviation industry, in addition to health, economic, and broader environmental benefits. Failures in detection will instead produce environmental harm. Here the focus should be on specification, verification and validation of swarm operations to monitor and mitigate wildfires.

### (c) Pollutant detection using floating swarm sensors

Scenario: A swarm of hundreds of small floating robots is deployed in water sources to sense pollutants and find their source. They communicate the state of the water source through their colour and position. This would allow people to make informed decisions about their interaction with the water source (swimming, fishing), as well as raise awareness about water quality [17].

Stakeholders: governments, water agencies, people using the water for leisure and/or sport.

Table 5 lists an outline of an ERA of a swarm for monitoring water pollution. Additional analysis of the risks and mitigations follows below.

**Table 5.** ERA for the deployment of a floating swarm to detect water pollutants.

category	ethical risk	level	mitigation
societal	loss of trust in the readout	M	the system will need to be thoroughly tested, verified and validated for false negative and false positive pollutant readouts which would undermine trust in the system.
societal	ownership and control of the robots is not clear	M	each robot should have a QR code with additional information.
environmental	swarm deployments are not sustainable	M	swarms should be made environmentally friendly by design (e.g. biodegradable, easy to collect, safe for wildlife and collision-benign). Aggregation of robots should be avoided to prevent robot-bergs.

#### (i) Societal risks

Loss of/inappropriate trust: The swarm will need to be robust in its signalling of pollutants, as errors may cause panic and false identification of sources of pollutants (false positives). Similarly, a lack of detection when pollutants are present could erode trust in the system (false negatives). Work on swarm specification, verification and validation could enable this, as well as oversight and frequent checking of the water through manual interventions and monitoring.

Lack of informed command: It may not be clear who is responsible for the command of the swarm or how to engage with it. This could be provided through clear information displayed around the water source and a QRcode on the robots, with information on whom to contact with questions or to report issues.

#### (ii) Environmental risks

Lack of environmental awareness (robots and operations): Hundreds to thousands of robots will need to be deployed in natural environments—making the robots themselves potential environmental hazards. It will be essential they are designed to be easy to retrieve and dispose of, and safe for wildlife (cannot be swallowed). The collective behaviour of the robots accumulating in certain areas may themselves cause a problem (robot-bergs, similar to fat-bergs in the sewage system). These should be avoided by programming the robots to stay apart.

### (d) Nanoswarms for cancer treatment

Scenario: Patients at risk of cancer are injected with a single preventative treatment of nanoswarm technology that monitors the body for signs of cancer long term and treats cancer cells when encountered.

Stakeholders: clinicians, regulators, patients, pharmaceutical industry, healthcare providers, cancer research organisations and cancer charities/non-governmental organisations.

Table 6 lists an outline of an ERA of nanoswarms for cancer treatment. Additional analysis of the risks and mitigations follows below.

**Table 6.** ERA for the deployment of nanoswarms for cancer treatment.

category	ethical risk	level	mitigation
individual	swarm causes physical harm to the patient	H	clinical trials are needed, along with a regulatory framework to assess safety of nanoswarm technology in the clinic.
individual	continuous surveillance of medical data	M	importance given to software solutions that securely store information related to medical records, with the option of keeping data private to the patient (not shared).
individual	misuse of the nanoswarm	M	misuse requires changing the design of the nanoswarm, each nanoswarm instantiation should therefor undergo its own ERA.
individual	inability to control the swarm once deployed	M	through clinician oversight, enable monitoring of swarm performance (e.g. through biomarkers) and a ‘stop and clear’ mechanism.
individual and environmental	nanoswarm toxicity	M	clinical and environmental trials to limit toxicity of the nanoswarm.
societal	swarm causes psychological harm to the patient	M	clear communication and consultation, continuous monitoring and clinician engagement to reassure the patient.
societal	inappropriate trust in the nanoswarm	M	clinical trials, clear communication and consultation are needed to manage expectations.
societal	treatment is costly for most patients	H	lessons learned from global vaccine distribution should be considered to increase access.
societal	patients lack the ability to continuously consent to their treatment	M	new mechanisms for dynamic consent should be adopted, including the ability to ‘stop and clear’ the swarm if consent is withdrawn.
societal	fear of technology prevents potential benefit	M	demystify the technology and improve public engagement and co-creation.

#### (i) Individual risks

Physical harm by the swarm: Unlike other swarm robotic deployments where individual robots may also cause harm, the impact of a single nanobot is possibly negligible at the scale of full-body deployments. Harm is mostly caused, therefore, by the interaction of trillions of nanobots in the body. This deployment poses the following two challenges: (i) demonstrating that the nanoswarm does no harm when cancer is not present, including indirectly (e.g. impacting the immune system) and (ii) selectively killing cancer cells, and sufficiently so as to be an effective treatment when they are present. To enable monitoring and prevent physical harm, a marker should be designed that signals the killing of cancer cells versus healthy cells, allowing for a readout that can be actioned (for example, sending a ‘stop and clear’ signal to the swarm after treatment is completed or if it is not operating as intended). In addition, a new framework for the regulation of nanoswarms in the clinic should be explored [34].

Lack of privacy and confidentiality: The long-term surveillance of the body for cancer cells could be seen as invasive, especially if the state of the system results in markers that can be monitored over time (e.g. through an app). Similar to challenges with long-term cancer screening, studies will be needed to determine the correct amount of information fed back to the patients to avoid unnecessary alarm (e.g. false positives), while still being transparent. Importance will be given to the privacy and security of the tools used to monitor the body. It could also be possible to not track data and allow the preventative measure to operate without external visible feedback.

Misuse: The technology could be misused, for example, by intentionally targeting healthy cells in the case of terrorism or hacking. Use of the technology will need to be regulated, like all drugs and medical devices.

#### (ii) Societal risks

Psychological harm by the swarm: Having a potential long-term invisible treatment may cause emotional harm if patients feel increased anxiety, fear or depression about whether treatments will work and what will happen in the future. Clear communication is needed throughout treatment to explain the way the swarm works and provide continuous monitoring and clinician engagement to reassure the patient.

Loss of trust/inappropriate trust: Patients may lose trust in the system if cancer still develops despite preventative treatment or if side-effects are present when cancer is not present. Similarly, they may overtrust the swarm—seeing it as a miracle worker. Like any medicine, the treatment will need to undergo clinical trials to assess its performance. Clear communication will be needed to manage expectations.

Inequality of access: The preventative treatment may be out of reach for most patients owing to cost and access. Building on lessons learned from vaccine deployment models, developing principles of equitable access throughout research, development, procurement, allocation, scale-up and distribution could be explored to increase capacity and provide global access.

Lack of informed consent: While consent may be given for the initial treatment, it is unclear how informed consent can be granted over long periods of time and in the presence of uncertainty. Newer models of consent are being proposed such as dynamic consent, which could be adopted [35–37]. It will also be important to have a mechanism to ‘stop and clear’ the swarm, so that if consent is ever withdrawn it can be actioned.

Lack of informed command: From the initial treatment, nanoswarms will operate autonomously, providing few mechanisms to command the swarm. This can be mitigated by enabling monitoring of the swarm performance and a ‘stop and clear’ command. It will also be important to allow information to be shared with a clinician for oversight and to train clinicians on this new technology.

#### (iii) Environmental risks

Lack of environmental awareness (robots and operations): The swarms will need to be safe inside and outside the body (disposal). This will be assessed through clinical and environmental trials.

Lack of environmental awareness (application): These swarms are an augmentation of the body’s function. Depending on public perception of these swarms, they may be seen as benign agents (such as vitamins) or akin to bodily pollution or cyborg technology. Importance will be given to demystifying the technology, public engagement and public consultation and co-design with stakeholders.

## 6. Discussion and conclusion

The four case studies set out above, across vastly different swarm applications, have highlighted salient ethical risks and mitigations that need to be actioned before swarms are deployed. Similar across all these assessments is the need for swarms to be co-created with stakeholders, safety of the swarm and predictability of its operations, clear and acceptable consent and command of the swarm, privacy and security protection, and the environmental impact of the swarms.

The mitigations articulated in the case studies have also highlighted opportunities where swarm engineering may lead to more ethical systems by design (distributed nature, local ownership and scale) but also where further research is needed to action the mitigations proposed.

On the positive side, distributed control seems to favour privacy and security of data, as information is processed at the edge [38]. Such distributed control is also local, potentially giving ownership, command and control to local communities. The scalability of swarms to huge numbers opens up environmental applications at scale, such as monitoring large areas, powering local logistics and mitigating fires, which may have a positive environmental impact [39].

Further research is needed in other aspects of swarm engineering that would enable important ethical risk mitigations. Many of these are reviewed in work by Wilson *et al*. [40] on trustworthy swarms.

First, the distributed nature of swarms, their emergent properties and scale mean advances are needed in how users can understand, monitor and control swarms. Work has focused on how the interface to swarm information affects human understanding of swarm behaviour and cognitive workload. Strategies include varying the viewpoint [41] and presenting information in more intuitive ways, for example, through heat maps displaying the confidence of received information [42], augmented reality systems and gesture-based swarm control through haptic interfaces [43–45] and interfaces that display the percentage of swarm distribution [46]. Control of swarms is also an open question, made challenging by our inability to easily handle multiple robots [47]. One question is which level of control is appropriate, from fully autonomous to full control by human operators or hybrid solutions [48,49]. Reducing human input can sometimes improve performance of the system, so-called ‘neglect benevolence’ [50]. However, when used well, human input can also be central to effective swarm operation [51]. New mechanisms of control are also explored, including ‘tangible’ user interfaces that make use of gesture, voice or physical touch [45]. Others use augmented reality to improve human–swarm interactions [52]. Swarms can also be controlled through different mechanisms, including providing swarm-level commands or taking control of individual robots [53]. Artificial evolution has been explored to automatically simplify the control for human operators by allowing them to focus on high-level commands at the swarm level [54,55].

Second, new mechanisms are needed to build confidence in the operation of swarms and their emergent properties, including to specify, verify and validate swarm behaviour, and make them reliable. Early work to mathematically model and hence verify the behaviour of swarm robotic systems made use of probabilistic finite-state models [56,57]. Recent research by Abeywickrama *et al*. [58] introduces assurance of emergent behaviour in autonomous robotic swarms, a novel process for ensuring the safety of emergent behaviours in autonomous robotic swarms. Fault detection has been explored, inspired by the immune system [59–61], blockchain technology to identify byzantine robots [62] or data-driven methods [63].

Third, more work is needed to engage with the stakeholders of swarm technology to understand which swarms should be deployed and how. Recent work has used mutual shaping and co-design techniques to identify use-cases for swarms in firefighting, warehousing and inspection [64–66]. More work is needed, however, with experts in social science and human factors, to design swarm technology.

In conclusion, this work proposes—for the first time—a framework for the ethical governance of swarm robotic systems that considers the unique emergent properties of swarms, their opportunities for scale, robustness and adaptability, and challenges in human need to monitor, control and build trust in these systems. The framework adds a layer of additional ethical governance that comprises the following six processes: (i) stakeholder consultation and governance, (ii) ERA, (iii) human–swarm interaction for monitoring and intervention, (iv) data logging, (v) accident/incident investigation, and (vi) end-of-life re-purposing and/or re-cycling. We have developed four use case studies in city logistics, firefighting, water pollutant monitoring and cancer treatment to highlight the very different and salient tensions that underpin the need for our framework in real-world applications. Our aim is to set out the foundations of this new ethical governance framework early, allowing for refinement and iterations to the framework as swarms are increasingly deployed in the real-world over the next decade.

## Data Availability

This article has no additional data.

## Footnotes

^1^
Also known as human-swarm teaming.

^2^

https://ec.europa.eu/futurium/en/ai-alliance-consultation/guidelines/1.html

^3^

https://oecd.ai/en/ai-principles

^4^
Which maps approximately to the ‘societal: fairness and justice‘ category of table 1.

^5^
In aviation, there is strong evidence that the thorough investigation of near-misses has significantly improved safety [28].

^6^
Perhaps the unexpected behaviours are localized to a small cluster of robots in which case those robots can be extracted while the rest of the swarm continues to operate.

^7^
If the application domain is one that is regulated, then, in the event of a serious accident the regulator is likely to initiate the accident investigation.
